# The Choice of Local Treatment Modalities for Patients with Brain Metastases from Digestive Cancers

**DOI:** 10.1155/2019/1568465

**Published:** 2019-11-21

**Authors:** Jun Dong, Liyan Wu, Fang Wang, Jinsheng Huang, Pili Hu, Bei Zhang, Liang-ping Xia

**Affiliations:** ^1^Department of Intergrated Therapy in Oncology, Sun Yat-Sen University Cancer Center, State Key Laboratory of Oncology in South China, Collaborative Innovation Center for Cancer Medicine, No. 651, East Dong Feng Road, Guangzhou, Guangdong 510060, China; ^2^Department of Clinical Medicine, Sun Yat-Sen University, No. 74, Zhong Shan Er Lu, Guangzhou, Guangdong 510060, China; ^3^Department of Oncology, The First Affiliated Hospital of Sun Yat-Sen University, No. 58, Zhong Shan Er Lu, Guangzhou, Guangdong 510060, China

## Abstract

**Background:**

Brain metastases (BMs) from digestive cancers are rare; therefore, no optimal treatment modality has been defined.

**Methods:**

We retrospectively reviewed the clinical data of 68257 patients with digestive cancers. Propensity score matching (PSM) was used to balance patient backgrounds between groups. Survival differences between different treatment modalities were compared. Univariate and multivariate Cox proportional hazards models were performed to identify prognostic factors on overall survival (OS).

**Results:**

270 patients with BM entered the study. In the entire group, the median survival time after diagnosis of brain metastases was 10.25 months (95% CI: 8.41–12.09 months); local treatment could significantly prolong OS (respectively, *P* < 0.01; even after PSM, *P* < 0.01); combination treatment was more effective than single treatment modality (respectively, *P* < 0.01; even after PSM, *P* < 0.01). However, each combination modality was identically effective (*P* > 0.05). When patients were divided into three groups based on 1, 2-3, or more than 3 metastatic lesion(s), same results were identified between local treatment and without local treatment (1 lesion, *P* < 0.01; 2-3 lesions, *P* < 0.01; more than 3 lesions, *P* < 0.01, respectively) and combination and single treatment (*P* < 0.01, *P*=0.02, *P*=0.03, respectively). However, there was no difference between different combined treatments (*P* > 0.05). Multivariate analysis revealed that performance status (*P* < 0.01), presence of extracranial metastasis (*P*=0.04), number of BM (*P* < 0.01), and local treatment for BM (*P* < 0.01) were independent prognostic factors.

**Conclusions:**

Regardless of the number of brain lesions, local treatment achieved higher overall survival times than no local treatment, and combination therapy could offer survival benefit to patients as compared with single therapy.

## 1. Introduction

As the most common intracranial tumors, brain metastases (BMs) occur in 20–40% of patients with cancer and are found most frequently in association with lung cancer (36–64%), breast cancer (15–25%), and melanoma (5–20%) [1]. Despite the high prevalence of the digestive cancers worldwide, BM from digestive cancers is a rare and late event with a reported incidence of 4–6% [2, 3]. The incidences of intracranial metastasis from different regions of the digestive system are as follows: esophagus (1.4–1.8%) [4], stomach (0.16–0.69%) [5], liver (1.3–2.9%) [6], gallbladder (<0.5%) [7], pancreas (0.1–0.3%) [8], and colorectum (1–4%) [9]. However, compared with brain metastases originating from the lung and breast, survival in patients suffering brain metastasis from digestive cancers was found to be diminished [10, 11]. The frequency of BM from digestive cancers has increased over time for several reasons, including greater patient awareness of symptoms for early diagnosis, advances in neuroimaging procedures, and more effective systemic treatments that prolong survival but do not cross the blood-brain barrier [12, 13].

Alternative therapeutic approaches to BM include surgical resection, whole brain radiotherapy (WBRT), and stereotactic radiosurgery (SRS). Surgical resection is considered as the main treatment of brain metastases in selected patients with 1 to 3 brain lesions, controlled systemic disease, and better performance status [14]. WBRT is often considered for patients with multiple metastases or short life expectancy since its side effects like cognitive deterioration. Compared to WBRT, SRS is now widely used for patients with four or fewer brain lesions in recent years due to improved cognitive outcomes and more favorable quality of life [13, 15]. There is evidence that even for patients with up to ten brain metastases, SRS might be appropriate [16]. Moreover, new techniques like HyperArc and Multiple Brain Mets are able to facilitate the use of SRS to treat multiple brain metastases in a single or few sessions [17]. WBRT is often considered for patients with multiple metastases or short life expectancy. Due to the lack of a general consensus on the optimal treatment of BM from digestive cancers, the treatment strategy remains often tailored in a multidisciplinary context. Currently, most previous studies are retrospective single-center analysis of clinical characteristics, predictive factors, and prognostic factors in sample size limited cohorts, and very few reports have evaluated the outcomes of various treatment modalities. However, we are still confused to make choice on the optimal treatment strategy.

The aim of the present study was to retrospectively analyze the clinical features of 270 patients with BM from 68257 digestive cancers, compare the outcomes of various treatment modalities, and identify prognostic factors to guide clinicians to choose optimal treatment for patients with BM from digestive cancers. Because there were background intergroup differences and a large discrepancy in the number of patients in different groups, propensity score matching was conducted. To the best of our knowledge, this is the first retrospective study which conducted propensity score matching and evaluated the outcomes of various treatment modalities for BM from digestive cancers. It also presents the largest number of patients with BM from digestive cancers analyzation to date.

## 2. Methods

### 2.1. Patient Population

Between April 1989 and January 2018, patients with BM from digestive cancers treated at the Sun Yat-sen University Cancer Center and the First Affiliated Hospital of Sun Yat-sen University were included in this study. Database examination was used to following identification of patients.

This present study was compatible with the basic standards of the Declaration of Helsinki and was approved by the Institutional Review Board (IRB-approved number, YB2018-67) of Sun Yat-sen University Cancer Center. The methods were carried out in accordance with the approved guidelines.

Inclusion criteria were as follows:

1. Primary malignancy arising from the esophagus, stomach, liver, gallbladder, pancreas, colon, rectum or anus, or an unknown primary tumor location but histopathologic tissue evidence that the tumor arose from digestive cancers
2. Primary malignancy confirmed by histopathologic tissue diagnosis or/and computed tomography (CT) or/and magnetic resonance imaging (MRI)
3. Brain metastasis diagnosed by means of MRI or/and CT with or without surgical pathology

Exclusion criteria were as follows:

1. Patients with incomplete information
2. Patients with multiple tumor primaries (i.e., lung and esophagus)

### 2.2. Definitions

Synchronous brain metastasis was defined as brain metastasis diagnosed before or at the time of or within 30 days after primary digestive cancer diagnosis. However, subsequent brain metastasis included brain metastasis diagnosed more than 30 days after the primary tumor diagnosis. The brain metastasis-free interval was defined as the time interval from primary disease diagnosis until brain metastases. Overall survival (OS) was calculated from the date of BM diagnosis to the date of death or the date of last follow-up. Median overall survival (mOS) is the amount of time after which 50% of the patients have died and 50% have survived.

### 2.3. Variables

The medical records of all patients were reviewed to obtain the following factors: (i) patient demographics including gender, age, and ECOG performance status (PS) score at initial diagnosis of digestive cancer, date of primary disease diagnosis, primary disease site, primary histology (hepatocellular carcinoma, squamous cell carcinoma, adenocarcinoma, others including undifferentiated carcinoma, adenosquamous carcinoma, esophagus small cell carcinoma, cholangiocarcinoma, mixed cell carcinoma, and hepatoblastoma), the presence of extracranial metastasis and type of treatment for primary tumor; (ii) BM characteristics including date of BM diagnosis, synchronous or subsequent brain metastasis, number of BM (1, 2, 3, ≥4), type of treatment for BM (surgical resection, WBRT, SRS), and date of death.

### 2.4. Treatment Modalities

The treatment strategy for BM was designed by neurosurgeons and/or radiation oncologists according to the patient's general condition, the location and number of BM, the presence of extracranial metastases. Patients were classified into seven treatment groups; no local treatment; SRS alone; surgery alone; WBRT alone; SRS plus surgery; SRS plus WBRT; surgery plus WBRT. Generally, patients with a single BM, located near an important region, symptomatic mass effect, or massive edema are most likely to benefit from neurosurgical resection. The decision for surgical resection was based on tumor size, location, and associated symptoms. Then the surgery was performed by experienced neurosurgeons using standard technique. SRS is a better option for patients with one to four brain metastases, no larger than 4 cm in diameter, located at gray-white junction. SRS was performed with linear accelerators or gamma knife. The doses typically used are 16 to 20 Gy. WBRT is often considered in cases that the principal treatment for patients with multiple metastases, oligometastases of large size, poor PS, or recurrence after surgery or SRS. WBRT was performed with 6-megavoltage (MV) linear accelerator, using opposed lateral fields. The most common fractionation regimens were 30 Gy/10 fractions.

### 2.5. Follow-Up

Patients were followed up after BM diagnosis generally consisted of a clinical examination and CT or MRI imaging at three-to-six-month intervals according to routine institutional practice. Patients were followed until death or loss to follow-up.

### 2.6. Propensity Score Matching

For the comparison of the clinical outcomes of various treatment modalities, potential confounding and selection biases may exist because the treatments were not randomly assigned in this patient population. Propensity score matching was used to make the results more detailed and reliable. The propensity score was calculated in a logistic regression model with 9 parameters: age, gender, performance status, primary disease site, primary histology, radical surgery for primary tumor, presence of extracranial metastasis, synchronous or subsequent brain metastasis, and number of BM. This study used a 1 : 1 optimal matching without replacement. The matching process was performed with EmpowerStats (http://www.empowerstats.com/).

### 2.7. Statistical Analysis

Variables were analyzed for descriptive statistics as appropriate. Comparisons of patient and tumor characteristics were performed using the Fisher exact test and Pearson *χ*^2^ test where appropriate. The Kaplan–Meier method was used to calculate overall survival and generate survival curves. The log rank tests were used to compare survival differences between different treatment modalities. Univariate and multivariate Cox proportional hazards models were performed to explore the effect of independent variables on overall survival. All statistical analyses were performed using the statistical software SPSS version 20 (SPSS Inc, Chicago, IL). A *P* value ≤0.05 was considered statistically significant.

## 3. Results

### 3.1. General Characteristics

A total of 68257 patients underwent treatment for digestive cancers in these two institutions; 270 (0.40%) who met the inclusion criteria were retrospectively analyzed. There were 45 patients with esophageal cancer, 44 with gastric cancer, 56 with liver cancer, and 125 with colorectal cancer. The total incidence of BM were 0.40%, and 0.38%, 0.39%, 0.23%, and 0.61% in patients with esophageal cancer, gastric cancer, liver cancer, and colorectal cancer, respectively (*P* < 0.01) (Table 1). In the entire patient cohort with BM, most patients had extracranial metastases (71.9%) and subsequent brain metastases (76.3%). 203 patients (75.2%) were men. The median age at initial diagnosis of digestive cancers was 58 years (range, 21–85). 140 patients (51.9%) had one brain metastasis only; 44 (16.3%) had two lesions; 15 (5.6%) had three, and 71 (26.3%) had more than 3 brain metastases. 99 patients (36.7%) did not receive local treatment for brain metastases; 44 patients (16.3%) were treated with SRS alone; 50 patients (18.5%) were treated with surgery alone; 40 patients (14.8%) were treated with WBRT alone; 15 patients (5.6%) were treated with SRS plus surgery; 12 patients (4.4%) were treated with SRS plus WBRT; 10 patients (3.7%) were treated with surgery plus WBRT. Patients lacking local treatment for BM got higher PS score compared to all other groups (*P* < 0.01). There was no significant difference in the distribution of patients across all groups based on gender, age, and presence of extracranial metastasis, as shown in Table 2.

### 3.2. Survival Data in the Entire Patient Cohort

The median follow-up was 27.07 months with a range of 0.30–184.28 months. At the time of the last follow-up, 112 patients (41.5%) were alive and 158 patients (58.5%) had died. 78 (78.8%) of 99 patients died in the no local treatment group; 23 (52.3%) of 44 patients died in the SRS alone group; 23 (46.0%) of 50 patients died in the surgery alone group; 26 (65.0%) of 40 patients died in the WBRT alone group; 1 (6.7%) of 15 patients died in the SRS plus surgery group; 3 (25.0%) of 12 patients died in the SRS plus WBRT group; and 4 (40.0%) of 10 patients died in the surgery plus WBRT group. Median brain metastasis-free interval in our series was 16.66 months. Median survival time for the entire cohort was 10.25 months (95% CI: 8.41–12.09 months). The survival rates at 6 months and 12 months were 63.2% and 42.4%, respectively. Median survival time was 14.09 months (esophagus), 10.74 months (gastric), 12.78 months (liver), and 11.60 months (colorectum), respectively. Brain metastasis-free interval and survival date according to primary tumor site are listed in Table 1.

### 3.3. Survival Date according to Treatment Subgroup

**Figure 1:**
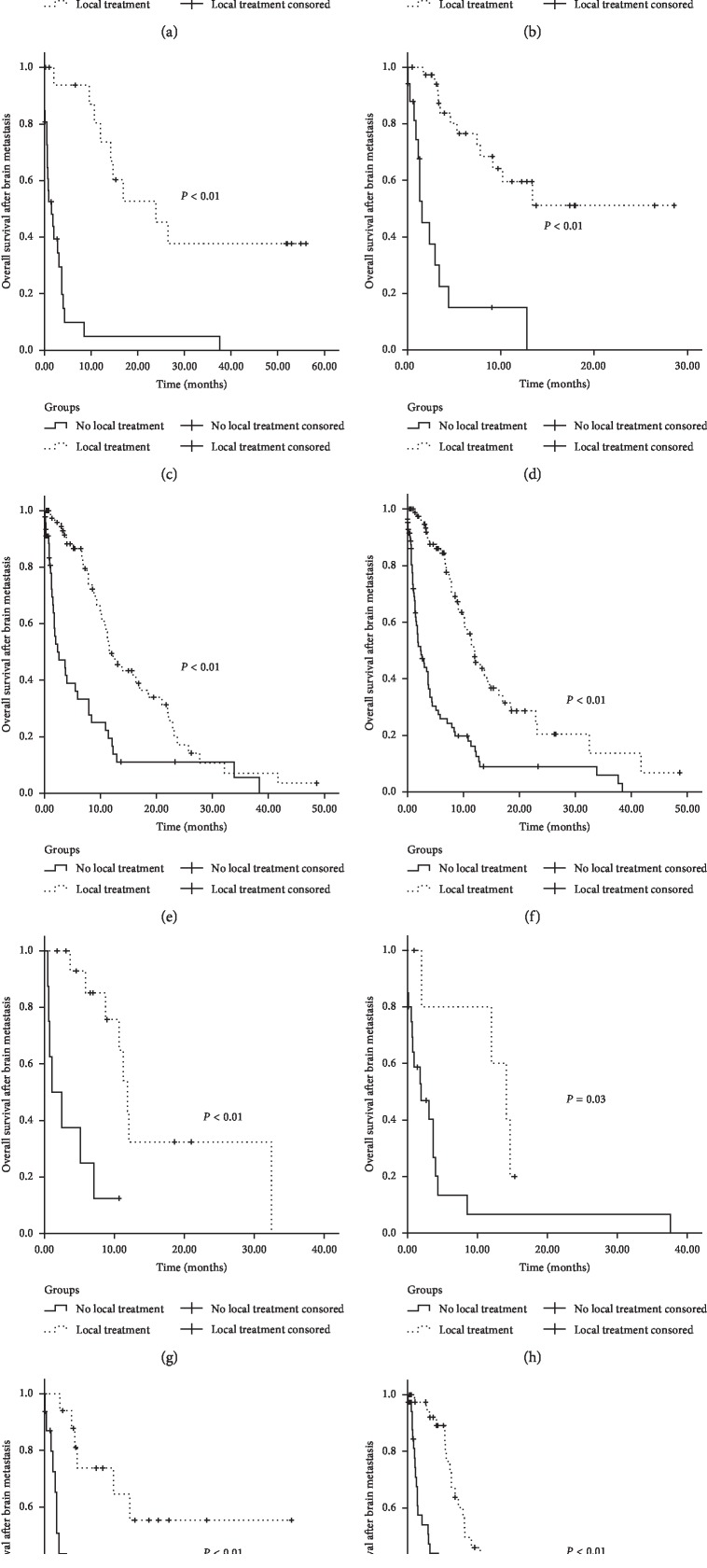
Kaplan–Meier overall survival curves compared patients without local treatment versus patients with local treatment for brain metastases. (a) All patients. (b) All patients with esophageal cancer. (c) All patients with gastric cancer. (d) All patients with liver cancer. (e) All patients with colorectum cancer. (f) All propensity score-matched patients. (g) Propensity score-matched patients with esophageal cancer. (h) Propensity score-matched patients with gastric cancer. (i) Propensity score-matched patients with liver cancer. (j) Propensity score-matched patients with colorectum cancer.

**Figure 2:**
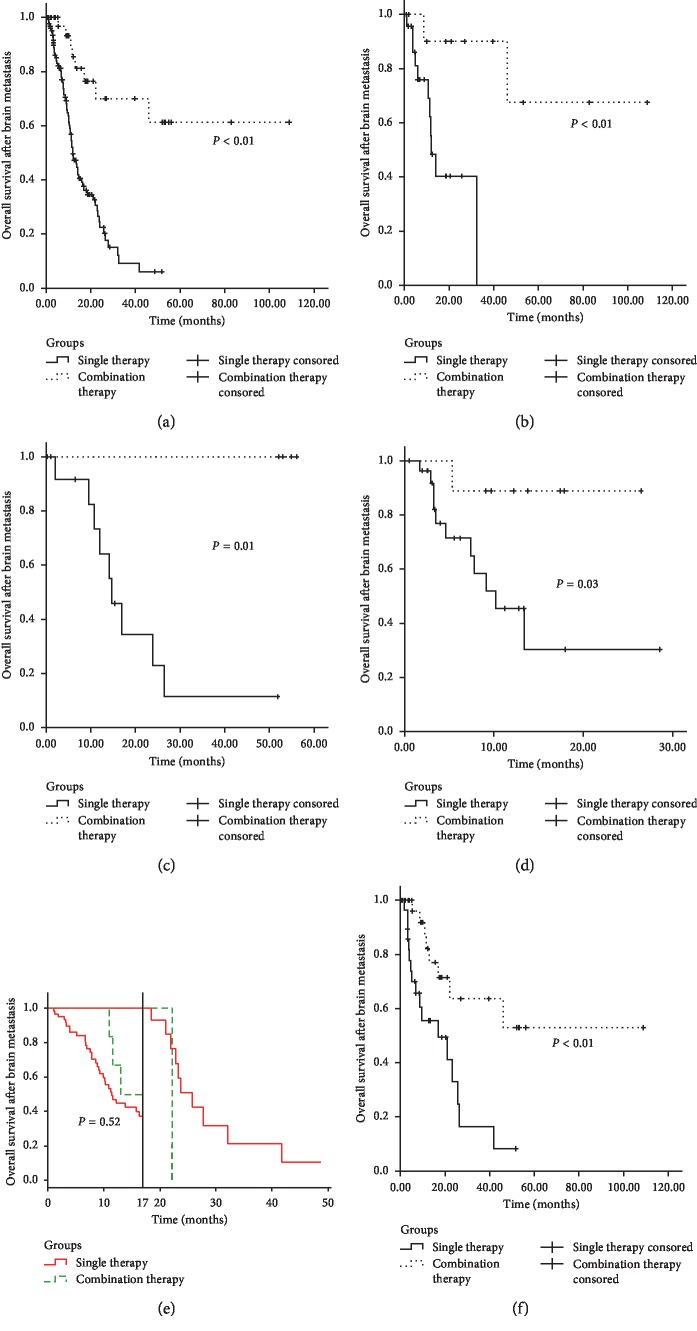
Kaplan–Meier overall survival curves comparing patients receiving single therapy versus combination therapy for brain metastases. (a) All patients. (b) All patients with esophageal cancer. (c) All patients with gastric cancer. (d) All patients with liver cancer. (e) All patients with colorectum cancer (as the log rank test is not a proper statistical inference method for two crossing survival curves, we did landmark analyses discriminating between events occurring before and after 17 months). (f) Propensity score-matched patients.

**Figure 3:**
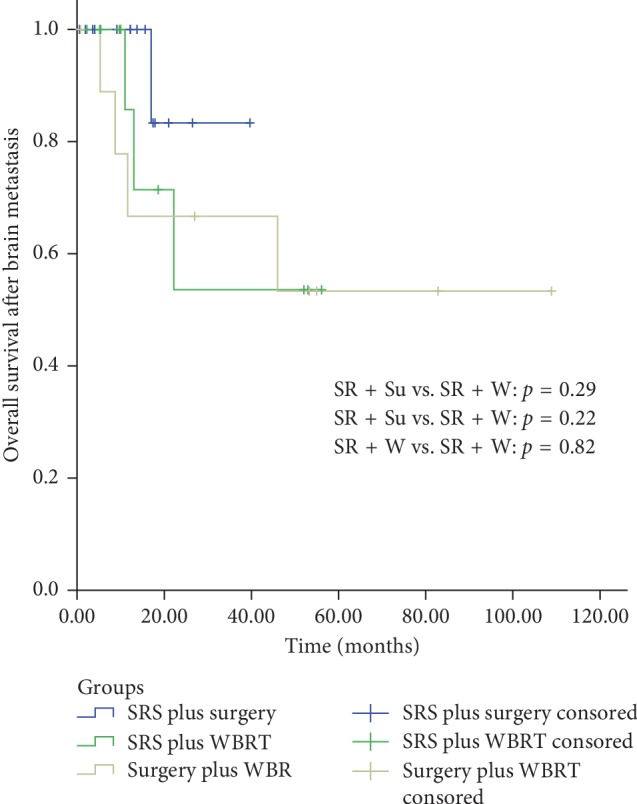
Kaplan–Meier overall survival curves according to combination therapy modalities for brain metastases.

**Figure 4:**
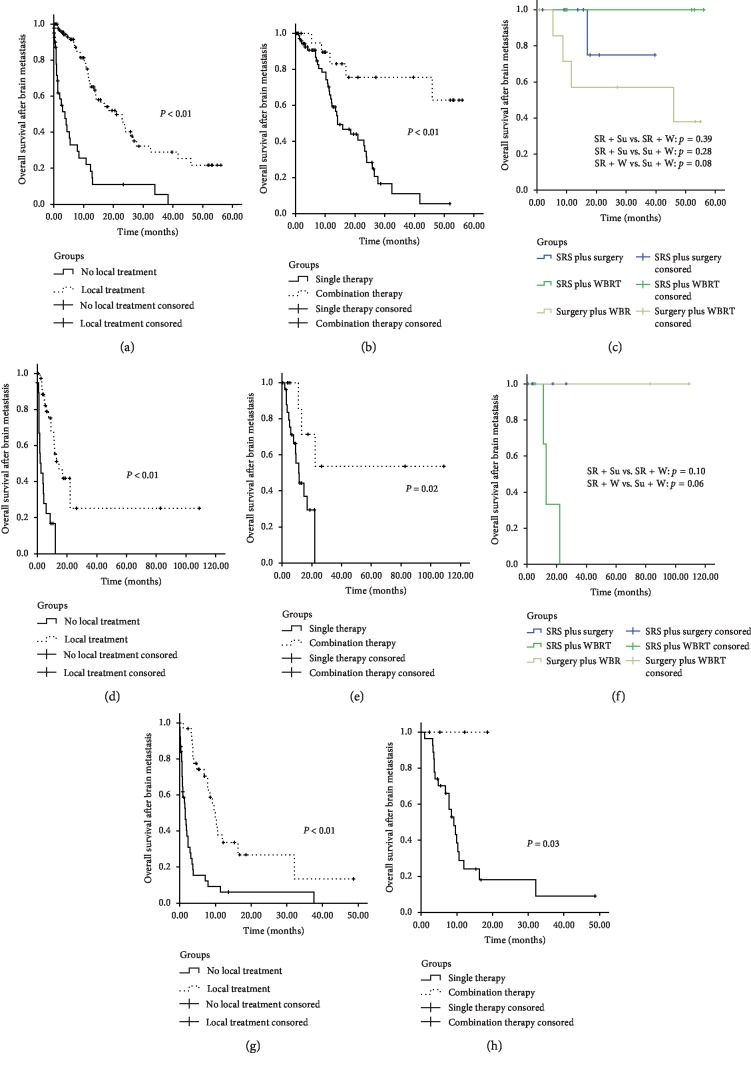
Kaplan–Meier survival curves for patients with a different number of brain lesions, according to treatment modalities for brain metastases. (a) For patients with one brain lesion, no local treatment versus local treatment. (b) For patients with one brain lesion, single therapy versus combination therapy. (c) For patients with one brain lesion, SRS plus surgery versus SRS plus WBRT, SRS plus WBRT versus surgery plus WBRT, and SRS plus surgery versus surgery plus WBRT. (d) For patients with two or three brain lesions, no local treatment versus local treatment. (e) For patients with two or three brain lesions, single therapy versus combination therapy. (f) For patients with two or three brain lesions, SRS plus surgery versus SRS plus WBRT and SRS plus WBRT versus surgery plus WBRT. (g) For patients with more than three brain lesions, no local treatment versus local treatment. (h) For patients with more than three brain lesions, single therapy versus combination therapy.

Patient prognosis varied greatly according to the treatment modality used (Figures 1–4). As shown in Figure 1(a), patients with local treatment achieved higher OS times than those patients without local treatment (*P* < 0.01). The 6-month and 12-month survival rates of patients without local treatment were 23.3% and 13.8%, respectively, with a median survival period of 1.97 months (95% CI: 1.13–2.81 months), and those of patients with local treatment were 84.7% and 58.0%, respectively, with a median survival period of 14.65 months (95% CI: 10.04–19.27 months). Similar results were obtained in the esophageal cancer, gastric cancer, liver cancer, and colorectal cancer subsets (Figures 1(b)–1(d)). Patient demographics and clinical characteristics for all patients (*n* = 270, *n* = 99 no local treatment and *n* = 171 local treatment) as well as propensity score-matched patients (*n* = 168, *n* = 84 no local treatment and *n* = 84 local treatment) are summarized in Table 3. After adjustment for propensity scores, except primary disease site (*P*=0.02), all covariates were well balanced among patients treated with and without local treatment. On propensity score-matched analysis, local treatment remained associated with improved OS compared with no local treatment (median OS 11.86 vs 2.46 months; *P* < 0.01) (Figure 1(f)).

Combination therapy group included patients from “SRS plus surgery group,” “SRS plus WBRT group,” and “surgery plus WBRT group.” Figure 2 shows the comparison results of single and combination therapy. Significantly better 6-month and 12-month survival rates were found for combination therapy (96.7% and 85.4%, respectively) compared to single therapy (81.1% and 49.7%, respectively) (*P* < 0.01). In the esophageal cancer, gastric cancer and liver cancer subsets, the difference between these median survival rates was statistically significant (better in combination therapy; Figures 2(b) and 2(d)).  Figure 2(e) presents the clinical results in patients with BM from colorectal cancer and the landmark analysis of events occurring within and after 17 months. Although two survival curves were separate intuitively before 17 months, this difference was not statistically significant (*P*=0.52). Because there were only 2 patients in the combination therapy group after 17 months, outcomes could not compare with the single therapy group statistically. Propensity score matching was performed to characterize the survival outcomes in single therapy group and combination therapy group with matched baseline characteristics. Patient demographics and clinical characteristics were well balanced between groups (Table 4). Propensity score-matched analysis showed that combination therapy achieved better survival than single therapy (*P* < 0.01; Figure 2(f)). As for which combination therapy is the best, this study did not find the significant difference in overall survival among SRS plus surgery, SRS plus WBRT, and surgery plus WBRT (Figure 3). Therefore, there is need for further studies with larger number of patients to be conducted.

It was stated in some papers that the number of brain lesions had a significant impact on the formulation of treatment strategy for BM [18–20]. To further demonstrate this viewpoint, 270 patients were stratified by the number of brain lesions, and the results are shown in Figure 4. For patients with a single lesion, the median overall survival was 3.68 months in the patients without local treatment versus 20.99 months in the patients with local treatment (*P* < 0.01; Figure 4(a)). Compared to single therapy, combination therapy could significantly prolong the overall survival of patients with single lesion (*P* < 0.01; Figure 4(b)). Comparing three different combination therapy methods (SRS plus surgery, SRS plus WBRT, and surgery plus WBRT), there was no significant difference in overall survival of patients with a single lesion (Figure 4(c)). Similar results were obtained in patients with 2 to 3 lesions and patients with more than 3 lesions: local treatment could offer survival benefit to patients compared with no local treatment (Figures 4(d) and 4(g)); combination therapy could offer survival benefit to patients comparing with single therapy (Figures 4(e) and 4(h)). In this study, the number of patients with more than one lesion and undergoing combination therapy is too limited to further analyze.

### 3.4. Prognostic Factors of Survival

Results of univariate and multivariate analyses are presented in Table 5. Using the Cox proportional hazards models, the following factors were found to be significant univariate prognostic factors of survival: performance status (*P* < 0.01), primary disease site (*P*=0.05), radical surgery for primary tumor (*P*=0.01), presence of extracranial metastasis (*P* < 0.01), synchronous or subsequent brain metastasis (*P*=0.05), number of BM (*P* < 0.01), and local treatment for BM (*P* < 0.01). Multivariate analysis revealed that performance status (*P* < 0.01), presence of extracranial metastasis (*P*=0.04), number of BM (*P* < 0.01), and local treatment for BM (*P* < 0.01) were independent prognostic factors.

## 4. Discussion

Brain metastases generally originate from lung cancer, breast cancer, melanoma, or renal cell carcinoma. Viewed as a late step in the course of disease, the morbidity of brain metastases from digestive cancers is rarely low. The literature recently proves that the site and histology of the primary cancer can influence the response rate of brain metastases to chemotherapy and radiation and result in clinical outcomes in patients with brain metastases [21–24]. Because lung cancer is the predominant primary cancer in nearly all brain metastases trials, it remains unclear whether data from these studies could be generalized for patients with BM from digestive cancers. In addition, there is no general consensus on the optimal treatment of BM from digestive cancers. This study retrospectively analyzed the clinical features, the effects of various treatment modalities on survival and prognostic factors of 270 patients with BM from 68257 patients with digestive cancers. In this series, the median age at initial diagnosis of digestive cancers was 58 years (range, 21–85 y). The majority of patients (76.3%) developed BM more than 30 days after the primary tumor diagnosis. 194 patients (71.9%) had extracranial metastases, and similar observations were described by other authors [25–27]. Data further showed that only 26.3% patients had multiple cerebral lesions (more than 3), which was different from lung cancer [1].

The brain metastasis-free interval highly depends on the primary site [28, 29]. In our series, median brain metastasis-free interval was 16.66 months (rang 0–173.01), which was much longer than that of lung cancer (usually within 1 year) [27, 30]. Whilst brain metastases from digestive cancers are rare, the clinical outcome of patients who develop them is poor. Our study showed that the survival time for patients without local treatment for brain metastases was 1.97 months, which was similar to that of reports in literature [10, 27]. Median survival time for the entire cohort was 10.25 months, which seemed to be longer than that reported previously (usually within 7 months) [8, 10, 18, 25, 27]. This may be a result of better patient awareness of symptoms and the application of screening programs leading to earlier diagnosis, effective systemic therapies, and aggressive local therapy. Our study included more new cases: patients who were enrolled by these studies were diagnosed with BM before January 2013, while 148 patients (54.8%) in our series were diagnosed with BM after 1 January 2013. Besides, there were 69.3% patients in our series with pretreatment PS scores of 0 or 1, which may offer patients more choices on treatment strategy. There was evidence that survival of patients with brain metastases of all pathological types appeared to be improving over time [11]. Data further showed that patients with BM from esophagus cancer achieved higher overall survival times than others (*P* < 0.01). Truskett [31] reported the survival time of patients with BM from esophagus primary received surgery (with or without radiation therapy) averaged up to 15 months. Weinberg et al. [32] demonstrated a median survival time of 26.2 months in the esophagus cancer patients with pretreatment KPS scores of ≥70. In our esophagus cancer subset, 4 patients with PS scores of 1 had lived more than 40 months after the date of BM diagnosis (overall survival time = 46.00, 53.25, 82.79, and 108.88 months, respectively). All of them underwent surgery plus WBRT for brain lesions.

Due to the lack of a general consensus on the optimal treatment, decision-making for the appropriate treatment of brain metastases is difficult. The median survival time after diagnosis of brain metastases was 12.22 months for patients who received SRS alone, 15.84 months for patients who received surgery alone, and 10.65 months for patients who received WBRT alone. The median overall survival time for combination therapy group was not reached, while the survival rates at 6 months and 12 months were 96.7% and 85.4%, respectively. Since the prognosis still remains poor, it is significant to choose an optimal treatment strategy in consideration of patient's general condition, characteristics of available treatment options, and the quality of life, such as cognitive and motor functions. From the present study, it seemed that local treatment associated with improved OS compared with no local treatment as well as combination therapy could offer survival benefit to patients as compared with single therapy, no matter what the number of brain lesions and the primary disease site are. But the present study did not address whether combination therapy would increase the risk of long-term toxicity and cognitive problems. Randomized controlled trials evidences [24, 33–35] showed that adjuvant WBRT improved intracranial control of disease without a survival advantage, whilst also resulted in more frequent cognitive deterioration compared with SRS alone. In addition, bevacizumab-based therapy was found to offer encouraging efficacy and acceptable safety for patients with brain metastases from non-small cell lung cancer [36, 37]. Finkelmeier et al. [38] reported that bevacizumab in combination with chemotherapy was a feasible option for patients with colorectal BM with a good safety profile. To find whether bevacizumab would become another treatment choice for patients with brain metastases from digestive cancers, more researches are needed.

We also demonstrated that performance status, presence of extracranial metastasis, number of BM, and local treatment for BM were independent factors affecting patient prognosis. Silva et al. [25] reported that once digestive cancers metastasize to the lung, the risk of subsequent metastasis to the brain is high, which was similar to the finding of Lin et al. [27].

There were several limitations to our study. First of all, as with all retrospective studies, interpretation of our outcomes is limited by potential selection bias, although we conducted propensity score matching to minimize it. Furthermore, the numbers of patients in several subgroups was small, and the heterogeneity of the population might make the findings difficult to generalize to other demographic groups. Nevertheless, given the rarity of patients with BM from digestive cancers and the necessity of intermediate intervention, a prospective randomized trial would be challenging to conduct. In addition, although several studies had reported the association between RAS or HER-2 or PIK3CA mutation and brain metastases from digestive cancers [39–43], the present study did not address the effects of genes mutations on brain metastases from digestive cancers since our cohort included many patients prior to the establishment of routine genetic analyses. Yet for all that, the present study has the largest number of patients with BM from digestive cancers analyzed to date, which also evaluate the outcomes of various treatment modalities in considerable detail.

## 5. Conclusions

In conclusion, regardless of the number of brain lesions, local treatment achieves higher overall survival times than no local treatment. Combination therapy could offer further survival benefit to patients compared with single therapy. These findings suggest that for patients with brain metastases derived from digestive cancers, aggressive local treatment allows for prolonged survival and combination therapy may be a preferred strategy. Single solitary tumor, PS score of 0 or 1, no presence of extracranial metastasis, and undergoing local treatment for brain metastasis significantly favored longer survival.

## Figures and Tables

**Table 1:** Site of primary tumor within gastrointestinal tract.

Primary disease site	No. of patients with digestive cancer	No. of patients with brain metastasis (%)	Median brain metastasis-free interval (months)	Median OS from diagnosis of BM (months)	6-month survival rates from diagnosis of BM (%)	1-year survival rates from diagnosis of BM (%)
Esophagus	11789	45 (0.38)	7.95	14.09	72.5	56.1
Gastric	11278	44 (0.39)	9.63	10.74	60.7	45.5
Liver	24566	56 (0.23)	21.44	12.78	59.8	53.0
Colorectum	20624	125 (0.61)	20.93	11.60	69.2	48.1
Total	68257	270 (0.40)	16.66	10.25	63.2	42.4

OS, overall survival; BM, brain metastasis.

**Table 2:** Patient demographics and clinical characteristics.

Characteristics	No local treatment, *N* = 99	Treatment modalities						Total *N* = 270	*P* value
		Local treatment *N* = 171							
		Single therapy, *N* = 134			Combination therapy, *N* = 37				
		SRS *N* = 44	Surgery *N* = 50	WBRT *N* = 40	SRS + surgery *N* = 15	SRS + WBRT *N* = 12	Surgery + WBRT *N* = 10		
Gender									
Male	68 (68.7%)	36 (81.8%)	35 (70.0%)	30 (75.0%)	12 (80.0%)	12 (100.0%)	10 (100.0%)	203 (75.2%)	0.06
Female	31 (31.3%)	8 (18.2%)	15 (30.0%)	10 (25.0%)	3 (20.0%)	0 (0.0%)	0 (0.0%)	67 (24.8%)	

ECOG performance score									
0/1	53 (53.5%)	36 (81.8%)	36 (72.0%)	29 (72.5%)	14 (93.3%)	10 (83.3%)	9 (90.0%)	187 (69.3%)	<0.01
2–4	46 (46.5%)	8 (18.2%)	14 (28.0%)	11 (27.5%)	1 (6.7%)	2 (16.7%)	1 (10.0%)	83 (30.7%)	

Age, years									
Median	58	57.5	57.5	54.5	52	66	60	58	0.33
Range	(21–85)	(30–79)	(35–76)	(30–81)	(23–72)	(24–74)	(30–67)	(21–85)	

Primary disease site									
Esophagus	10 (22.2%)	4 (8.9%)	9 (20.0%)	10 (22.2%)	3 (6.7%)	2 (4.4%)	7 (15.6%)	45 (100.0%)	<0.01
Gastric	26 (59.1%)	5 (11.4%)	6 (13.6%)	3 (6.8%)	0 (0.0%)	3 (6.8%)	1 (2.3%)	44 (100.0%)	
Liver	17 (30.4%)	12 (21.4%)	12 (21.4%)	6 (10.7%)	7 (12.5%)	1 (1.8%)	1 (1.8%)	56 (100.0%)	
Colorectum	46 (36.8%)	23 (18.4%)	23 (18.4%)	21 (16.8%)	5 (4.0%)	6 (4.8%)	1 (0.8%)	125 (100.0%)	

Primary histology									
Hepatocellular carcinoma	17 (17.2%)	12 (27.3%)	10 (20.0%)	5 (12.5%)	3 (20.0%)	1 (8.3%)	1 (10.0%)	49 (18.1%)	<0.01
Squamous cell carcinoma	10 (10.1%)	2 (4.5%)	5 (10.0%)	8 (20.0%)	1 (6.7%)	2 (16.7%)	5 (50.0%)	33 (12.2%)	
Adenocarcinoma	70 (70.7%)	28 (63.6%)	32 (64.0%)	24 (60.0%)	6 (40.0%)	8 (66.7%)	4 (40.0%)	172 (63.7%)	
Others	2 (2.0%)	2 (4.5%)	3 (6.0%)	3 (7.5%)	5 (33.3%)	1 (8.3%)	0 (0.0%)	16 (5.9%)	

Presence of extracranial metastasis									
No	24 (24.2%)	9 (20.5%)	16 (32.0%)	9 (22.5%)	8 (53.3%)	5 (41.7%)	5 (50.0%)	76 (28.1%)	0.08
Yes	75 (75.8%)	35 (79.5%)	34 (68.0%)	31 (77.5%)	7 (46.7%)	7 (58.3%)	5 (50.0%)	194 (71.9%)	

Radical surgery for primary tumor									
No	48 (48.5%)	10 (22.7%)	22 (44.0%)	7 (17.5%)	2 (13.3%)	0 (0.0%)	3 (30.0%)	92 (34.1%)	<0.01
Yes	51 (51.5%)	34 (77.3%)	28 (56.0%)	33 (82.5%)	13 (86.7%)	12 (100.0%)	7 (70.0%)	178 (65.9%)	

Synchronous or subsequent brain metastasis									
Synchronous	33 (33.3%)	9 (20.5%)	14 (28.0%)	7 (17.5%)	0 (0.0%)	0 (0.0%)	1 (10.0%)	64 (23.7%)	<0.01
Subsequent	66 (66.7%)	35 (79.5%)	36 (72.0%)	33 (82.5%)	15 (100.0%)	12 (100.0%)	9 (90.0%)	206 (76.3%)	

Number of brain metastasis									
1	42 (42.4%)	21 (47.7%)	45 (90.0%)	11 (27.5%)	8 (53.3%)	5 (41.7%)	8 (80.0%)	140 (51.9%)	<0.01
2-3	19 (19.2%)	15 (34.1%)	2 (4.0%)	12 (30.0%)	5 (33.3%)	4 (33.3%)	2 (20.0%)	59 (21.9%)	
≥4	38 (38.4%)	8 (18.2%)	3 (6.0%)	17 (42.5%)	2 (13.3%)	3 (25.0%)	0 (0.0%)	71 (26.3%)	

SRS, stereotactic radiosurgery; WBRT, whole brain radiotherapy; ECOG, Eastern Cooperative Oncology Group.

**Table 3:** Baseline characteristics of patients with and without local treatment.

Characteristics	Before PSM			After PSM		
	No local treatment *N* = 99	Local treatment *N* = 171	*P* value	No local treatment *N* = 84	Local treatment *N* = 84	*P* value
Age, years			0.66			0.87
Median	58	57		58	58	
Range	21–85	23–81		21–85	23–81	

Gender			0.06			0.73
Male	68 (68.7%)	135 (79.0%)		60 (71.4%)	63 (75%)	
Female	31 (31.3%)	36 (21.1%)		24 (28.6%)	21 (25%)	

ECOG performance score			<0.01			0.51
0/1	53 (53.5%)	134 (78.4%)		52 (61.9%)	57 (67.9%)	
2–4	46 (46.5%)	37 (21.6%)		32 (38.1%)	27 (32.1%)	

Primary disease site			<0.01			0.02
Esophagus	10 (10.1%)	35 (20.5%)		10 (11.9%)	16 (19%)	
Gastric	26 (26.3%)	18 (10.5%)		20 (23.8%)	6 (7.1%)	
Liver	17 (17.2%)	39 (22.8%)		16 (19%)	18 (21.4%)	
Colorectum	46 (46.5%)	79 (46.2%)		38 (45.2%)	44 (52.4%)	

Primary histology			0.12			0.69
Hepatocellular carcinoma	17 (17.2%)	32 (18.7%)		16 (19%)	15 (17.9%)	
Squamous cell carcinoma	10 (10.1%)	23 (13.5%)		10 (11.9%)	11 (13.1%)	
Adenocarcinoma	70 (70.7%)	102 (59.7%)		56 (66.7%)	53 (63.1%)	
Others	2 (2.0%)	14 (8.2%)		2 (2.4%)	5 (6%)	

Radical surgery for primary tumor			<0.01			0.64
No	48 (48.5%)	44 (25.7%)		35 (41.7%)	31 (36.9%)	
Yes	51 (51.5%)	127 (74.3%)		49 (58.3%)	53 (63.1%)	

Presence of extracranial metastasis			0.28			0.37
No	24 (24.2%)	52 (30.4%)		24 (28.6%)	18 (21.4%)	
Yes	75 (75.8%)	119 (69.6%)		60 (71.4%)	66 (78.6%)	

Synchronous or subsequent brain metastasis			<0.01			0.73
Synchronous	33 (33.3%)	31 (18.1%)		24 (28.6)	21 (25%)	
Subsequent	66 (66.7%)	140 (81.9%)		60 (71.4)	63 (75%)	

Number of brain metastasis			0.12			0.97
1	17 (17.2%)	32 (18.7%)		40 (47.6%)	43 (51.2%)	
2	10 (10.1%)	23 (13.5%)		12 (14.3%)	12 (14.3%)	
3	70 (70.7%)	102 (59.7%)		6 (7.1%)	5 (6%)	
≥4	2 (2.0%)	14 (8.2%)		26 (31%)	24 (28.6%)	

PSM, propensity score matching; ECOG, Eastern Cooperative Oncology Group.

**Table 4:** Baseline characteristics of patients receiving single therapy and patients receiving combination therapy.

Characteristics	Before PSM			After PSM		
	Single therapy *N* = 134	Combination therapy *N* = 37	*P* value	Single therapy *N* = 31	Combination therapy *N* = 31	*P* value
Age, years			0.66			0.55
Median	57	58		55	60	
Range	30–81	23–74		30–74	24–74	

Gender			0.03			0.70
Male	101 (75.4%)	34 (91.9%)		26 (83.9%)	28 (90.3%)	
Female	33 (24.6%)	3 (8.1%)		5 (16.1%)	3 (9.7%)	

ECOG performance score			0.07			1.00
0/1	101 (75.4%)	33 (89.2%)		27 (87.1%)	27 (87.1%)	
2–4	33 (24.6%)	4 (10.8%)		4 (12.9%)	4 (12.9%)	

Primary disease site			0.15			0.56
Esophagus	23 (17.2%)	12 (32.4%)		6 (19.4%)	10 (32.3%)	
Gastric	14 (10.5%)	4 (10.8%)		6 (19.4%)	3 (9.7%)	
Liver	30 (22.4%)	9 (24.3%)		6 (19.4%)	6 (19.4%)	
Colorectum	67 (50.0%)	12 (32.4%)		13 (41.9%)	12 (38.7%)	

Primary histology			0.05			0.42
Hepatocellular carcinoma	27 (20.2%)	5 (13.5%)		4 (12.9%)	5 (16.1%)	
Squamous cell carcinoma	15 (11.2%)	8 (21.6%)		4 (12.9%)	8 (25.8%)	
Adenocarcinoma	84 (62.7%)	18 (48.7%)		18 (58.1%)	16 (51.6%)	
Others	8 (6.0%)	6 (16.2%)		5 (16.1%)	2 (6.5%)	

Radical surgery for primary tumor			0.06			1.00
No	39 (29.1%)	5 (13.5%)		4 (12.9%)	4 (12.9%)	
Yes	95 (70.9%)	32 (86.5%)		27 (87.1%)	27 (87.1%)	

Presence of extracranial metastasis			<0.01			1.00
No	34 (25.4%)	18 (48.7%)		12 (38.7%)	12 (38.7%)	
Yes	100 (74.6%)	19 (51.4%)		19 (61.3%)	19 (61.3%)	

Synchronous or subsequent brain metastasis			<0.01			1.00
Synchronous	30 (22.4%)	1 (2.7%)		1 (3.2%)	1 (3.2%)	
Subsequent	104 (77.6%)	36 (97.3%)		30 (96.8%)	30 (96.8%)	

Number of brain metastasis			0.20			0.18
1	77 (57.5%)	21 (56.8%)		18 (58.1%)	18 (58.1%)	
2	25 (18.7%)	7 (18.9%)		6 (19.4%)	5 (16.1%)	
3	4 (3.0%)	4 (10.8%)		0 (0%)	4 (12.9%)	
≥4	28 (20.9%)	5 (13.5%)		7 (22.6%)	4 (12.9%)	

PSM, propensity score matching; ECOG, Eastern Cooperative Oncology Group.

**Table 5:** Univariate and multivariate analyses for brain metastasis.

	Univariable		Multivariable	
	HR (95% CI)	*P* value	HR (95% CI)	*P* value
Age, years (≥65 vs <65)	1.04 (0.74–1.46)	0.81	—	
Gender (male vs female)	1.40 (0.98–2.01)	0.08	—	
ECOG performance score (2–4 vs 0/1)	2.68 (1.92–3.76)	<0.01	2.31 (1.61–3.33)	<0.01
Primary disease site				
Esophagus vs liver	0.62 (0.34–1.12)	0.11	0.61 (0.33–1.11)	0.11
Gastric vs liver	1.27 (0.74–2.16)	0.39	0.90 (0.52–1.56)	0.70
Colorectum vs liver	1.20 (0.76–1.88)	0.43	0.68 (0.42–1.10)	0.11
Primary histology				
Hepatocellular carcinoma vs others	3.57 (1.07–11.87)	0.04		
Squamous cell carcinoma vs others	2.18 (0.63–7.47)	0.22		
Adenocarcinoma vs others	3.45 (1.10–10.85)	0.03		
Radical surgery for primary tumor (yes vs no)	0.66 (0.48–0.92)	0.01	1.15 (0.73–1.82)	0.55
Presence of extracranial metastasis (yes vs no)	1.77 (1.20–2.62)	<0.01	1.56 (1.03–2.36)	0.04
Synchronous or subsequent brain metastasis	0.71 (0.50–1.00)	0.05	0.79 (0.50–1.25)	0.32
Number of brain metastasis				
1 vs ≥4	0.42 (0.30–0.60)	<0.01	0.54 (0.37–0.79)	<0.01
2∼3 vs ≥4	0.57 (0.37–0.88)	0.01	0.70 (0.45–1.11)	0.13
Local treatment for brain metastasis (yes vs no)	0.21 (0.15–0.29)	<0.01	0.26 (0.18–0.38)	<0.01

HR, hazard ratio; CI, confidence interval; ECOG, Eastern Cooperative Oncology Group.

## Data Availability

The authenticity of this article has been validated by uploading the key raw data onto the Research Data Deposit public platform (http://www.researchdata.org.cn), with the approval RDD number as RDDA2018000866.
